# Sarcomatoid Variant of Urothelial Carcinoma (Carcinosarcoma, Spindle Cell Carcinoma): A Review of the Literature

**DOI:** 10.1155/2014/794563

**Published:** 2014-01-22

**Authors:** Anthony Kodzo-Grey Venyo, Sami Titi

**Affiliations:** ^1^North Manchester General Hospital, Department of Urology, Delaunays Road, Crumpsall, Manchester, UK; ^2^Royal Oldham Hospital, Department of Histopathology, Rochdale Road, Oldham, UK

## Abstract

*Background*. Sarcomatoid variant of urothelial carcinoma (SVUC) was added to the WHO classification in 2004. *Aims*. To review the literature. *Materials and Method*. Various internet databases were used. *Result*. SVUCs are rare biphasic malignant neoplasms exhibiting morphologic/immunohistochemical evidence of epithelial and mesenchymal differentiation with the presence or absence of heterologous elements. Some cases of SVUC have been associated with radiation therapy and cyclophosphamide treatment. Patients' ages range from 50 to 77 years (mean age 66). Patients tend to be younger and they more commonly presented with high-grade histology and advanced stage disease, in comparison with patients who had conventional urothelial carcinoma (CUC). Results of molecular/genetic studies strongly argue for a common monoclonal cell origin of both the epithelial and mesenchymal components in SUVC. The cancer specific survival of SVUC is poor in comparison with CUC. Radical surgical excision and chemoradiation may be associated with improved prognosis; chemoradiation as an organ preserving alternative to radical excision may be associated with improved outcome. There is no consensus opinion on the best treatment modalities for SUVC. *Conclusions*. SVUC is rare and is associated with inferior outcome compared with CUC. A multicentre trial of various treatment options is required. Cases of SVUC should be reported.

## 1. Introduction

Sarcomatoid variant of urothelial carcinoma (SUVC) is rare and because of its rarity most practitioners including urologists and oncologists may not have encountered the disease. The literature on sarcomatoid variant of urothelial carcinoma has been reviewed in the ensuing paper.

Sarcomatoid variant of urothelial carcinomas may generally present with specific symptoms of lower urinary tract symptoms and haematuria.

## 2. Literature Review

### 2.1. Definition

Sarcomatoid carcinoma is a fairly uncommon form of carcinoma whose malignant cells have histological, cytological, or molecular properties of both epithelial tumours (“carcinoma”) and mesenchymal tumours (“sarcoma”). Eble et al. [1] stated that sarcomatoid variant of urothelial carcinoma should be the terminology that is used for all biphasic malignant neoplasms which exhibit morphologic and/or immunohistochemical evidence of epithelial and mesenchymal differentiation with the presence or absence of heterologous elements acknowledged in the pathology report.

### 2.2. Associations

Lopez-Beltran and associates [2] stated that some of the patients with sarcomatoid variant of urothelial carcinoma have previous history of having been previously treated by means of radiotherapy or cyclophosphamide therapy.

### 2.3. Presentation

Lopez-Beltran et al. [2] iterated that some of the most frequent presenting symptoms and signs of sarcomatoid variant of urothelial carcinoma include haematuria, dysuria, nocturia, acute retention of urine, and lower abdominal pain. These aforementioned symptoms would generally be related to sarcomatoid urothelial carcinoma of the urinary bladder. In view of the fact that sarcomatoid urothelial carcinoma can affect the entire urothelium, it would be said that sarcomatoid urothelial carcinoma of the ureter and renal pelvis may present as loin pain and haematuria.

### 2.4. Incidence

Lopez-Beltran et al. [2] reported that sarcomatoid urothelial carcinoma usually presents between the ages of 50 years and 77 years and the mean age of presentation is 66 years. Black et al. [3] reported the incidence of sarcomatoid urothelial carcinoma to have ranged from 02% to 4.3%. The suggested incidence was confirmed in an analysis of the Surveillance, Epidemiology, and End Results (SEER) database. Wright et al. [4] reported that in total, 135 sarcomatoid urothelial carcinoma and 166 cases of carcinosarcoma were identified from a total of 182,283 patients with primary bladder cancer.

### 2.5. Macroscopic Appearance

**Figure 1:**
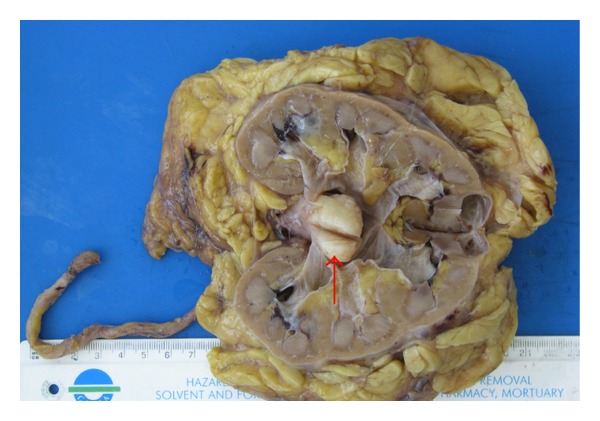
Right kidney with a big (45 mm) polypoid solid white tumour arising in renal pelvis (red arrow). This tumour was shown on microscopic examination to be a sarcomatoid variant of urothelial carcinoma.

Lopez-Beltran et al. [2] stated that sarcomatoid variant urothelial carcinomas often on macroscopic examination are seen as polypoid with large intraluminal masses (see Figure 1 which represents a nephroureterectomy specimen of a sarcomatoid variant of urothelial carcinoma encountered by the authors).

### 2.6. Microscopic Features

**Figure 2:**
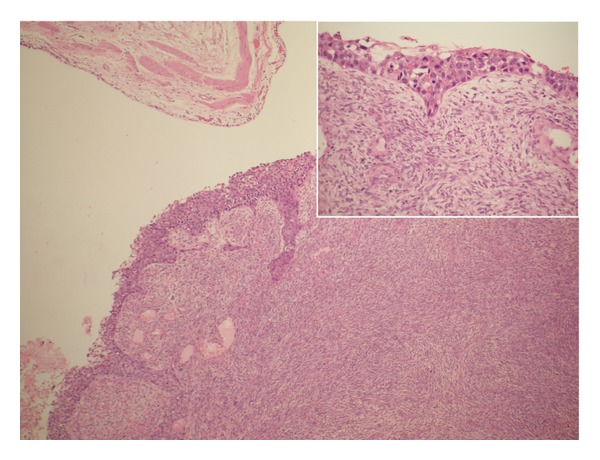
Low power magnification shows sarcomatoid variant of Urothelial Carcinoma of renal pelvis to be bulging into renal pelvis. Inlet: the covering urothelium with features of flat CIS.

**Figure 3:**
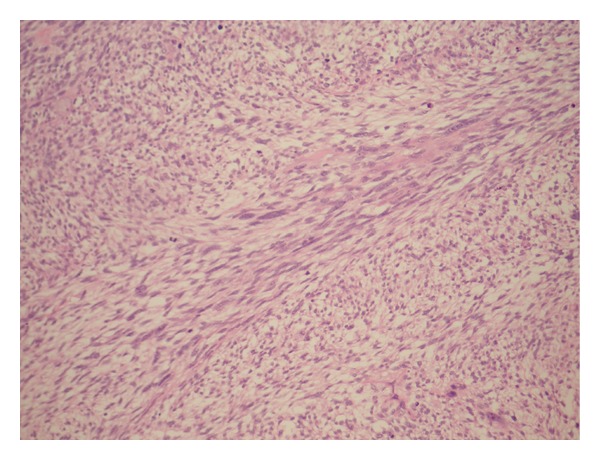
Sarcomatoid variant of urothelial carcinoma (sarcomatoid TCC of renal pelvis) is mainly composed of spindle cells, which appears focally to be arranged in a herringbone pattern.

**Figure 4:**
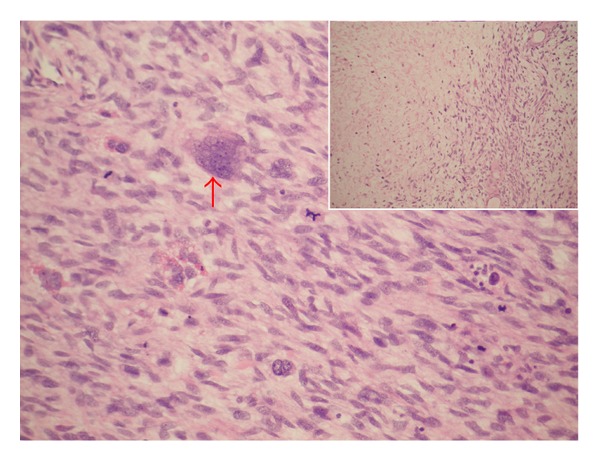
Sarcomatoid variant of urothelial carcinoma of renal pelvis (sarcomatoid TCC of renal pelvis) with multinucleated giant cell (red arrow) and numerous mitotic figures. Inlet: tumour necrosis on the left side.

**Figure 5:**
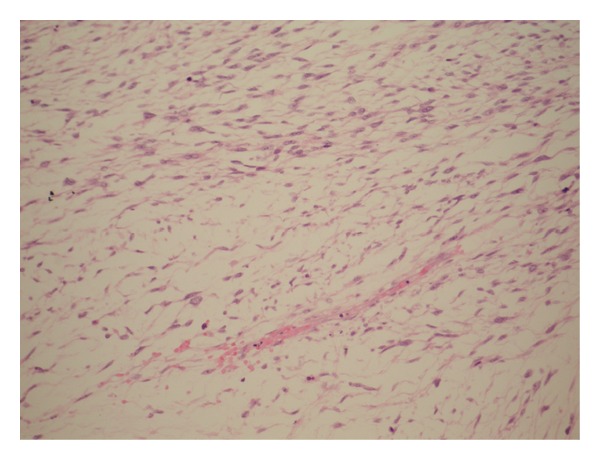
Sarcomatoid variant of urothelial carcinoma of renal pelvis (sarcomatoid TCC of renal pelvis) with evidence of focal myxoid changes haematoxylin and eosin staining ×10 magnification.

**Figure 6:**
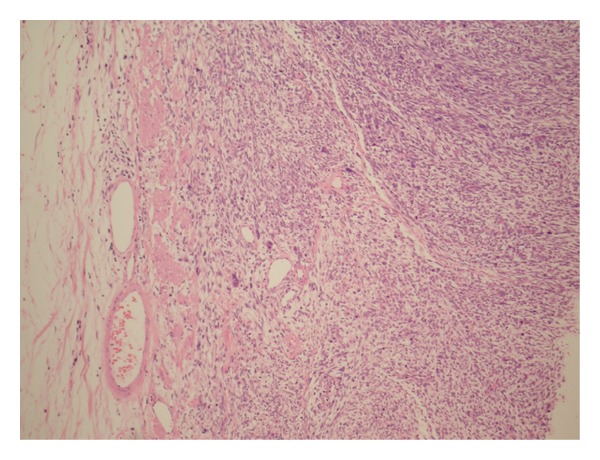
Sarcomatoid variant of urothelial carcinoma of renal pelvis, (“sarcomatoid TCC of renal pelvis”) with tumour extension beyond the muscularis propria haematoxylin and eosin staining ×10 magnification.

Lopez-Beltran et al. [2] stated that microscopically sarcomatoid urothelial carcinoma is composed of a urothelial, glandular, or small cell component which exhibit variable degrees of differentiation and that carcinoma in situ is found in 30% of cases of sarcomatoid variant of urothelial carcinoma and that occasionally carcinoma in situ is the only apparent epithelial component. A number of authors [2, 5–9] stated that a small subset of sarcomatoid variant of urothelial carcinoma of the urinary bladder and renal pelvis may exhibit a prominent myxoid stroma, a finding which may mislead the pathologist into making a diagnosis of inflammatory pseudotumour (inflammatory myofibroblastic tumour) but this is characteristically positive for anaplastic lymphoma kinase stains unlike sarcomatoid variant of urothelial carcinoma which is negative. Lopez-Beltran et al. [2] reported in their large case series that the most common heterologous element was osteosarcoma which was followed by chondrosarcoma but in addition multiple types may be present (See Figures 2; 3; 4; 5; 6 which show various microscopic features of a sarcomatoid variant of urothelial carcinoma of the renal pelvis encountered by the authors).

### 2.7. Immunohistochemical Features

**Figure 7:**
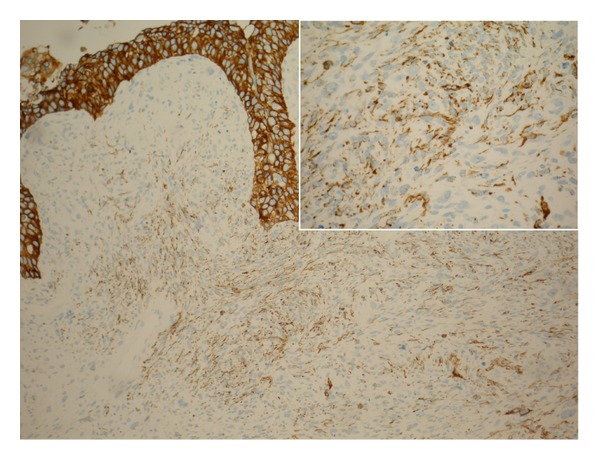
Sarcomatoid variant of renal pelvis (sarcomatoid TCC of renal pelvis) and flat CIS with positive immunohistochemical reaction for cytokeratin (MNF116). Inlet: immunohistochemistry reaction in tumour spindle cells.

**Figure 8:**
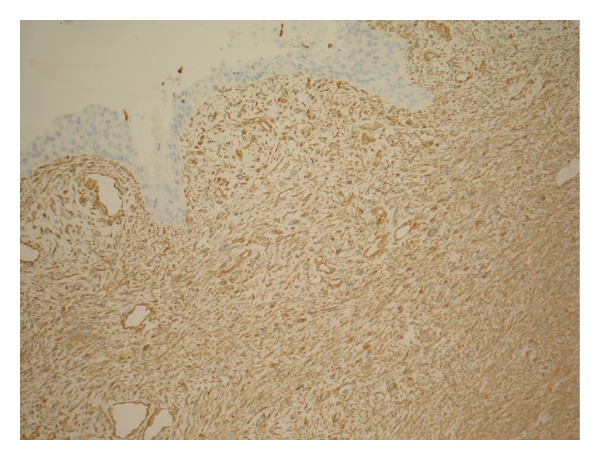
Sarcomatoid variant of urothelial carcinoma of renal pelvis (sarcomatoid TCC of renal pelvis). Immunohistochemicalstaining with Vimentin shows positive reaction in the underlying tumour spindle cells but negative in the overlying flat CIS.

It has been stated that immunohistochemical staining had revealed that epithelial elements react with cytokeratins; on the other hand, stromal elements react with specific markers which correspond with the type of mesenchymal differentiation (see Figures 7 and 8 showing positive immunohistochemical staining with cytokeratin (MNF16) and Vimentin taken from a sarcomatoid variant of urothelial carcinoma of renal pelvis encountered by the authors) [10, 11].

### 2.8. Genetic Studies

It has also been stated that results of molecular/genetic studies strongly argue for a common monoclonal cell origin of both the epithelial and mesenchymal components in sarcomatoid variant of urothelial carcinoma [10–12].

### 2.9. Treatment and Outcome

This rare biphasic variant of urothelial carcinoma had, until recently, only been described in small case series, which had suggested a poor outcome for patients with this variant of urothelial carcinoma [2]. Black and associates [3, 13] confirmed that patients with sarcomatoid variant of urothelial carcinoma have worse disease-specific and overall survival, even after adjusting for stage of tumour, in comparison with patients with high-grade pure urothelial cancer. No published articles have focused on treatment for sarcomatoid variant of urothelial carcinoma although one group suggested that radical cystectomy should be the preferred option of treatment for patients with stage T1 disease, rather than intravesical therapy [3, 13, 14].

The ensuing discussion details out some of the case reports and case series that have been published that relate to the presentation, investigation and diagnosis, and treatment and treatment outcomes of sarcomatoid variant of urothelial carcinoma.

## 3. Discussion and Narrations from Some of the Reported Cases

It has been stated that although sarcomatoid variant of urothelial carcinoma is rare, sarcomatoid carcinoma including carcinosarcoma is more common than primary sarcoma of the urinary bladder [2, 4, 6, 11, 15–21]. In 2009, Amin [15] stated that more than 100 cases of sarcomatoid variant of urothelial carcinoma had been reported in the literature at that time and that a recent SEER data analysis revealed that 301 cases were sarcomatoid carcinomas and carcinosarcomas among 46,515 patients (0.6%) of carcinoma of the urinary bladder. The terminology carcinosarcoma has been used by some authors, and in earlier publications, to designate tumours which had overt epithelial histology admixed with sarcomatous histology with heterologous elements. The latest World Health Organization (WHO) classification in 2004 acknowledged this controversy and endorsed the terminology sarcomatoid carcinoma as others have done [1, 20, 22].

Molecular studies by two groups of researchers [11, 12] revealed a common clonal origin for the carcinomatous and sarcomatous components, and the outcomes of tumours with and without heterologous elements are largely similar. Nevertheless, a recent study by Wright et al. [4] revealed that data which was reported to the SEER registry and analysed for outcome strictly on the basis of ICD-O-3 histological code showed that sarcomatoid variant of urothelial carcinoma and carcinosarcoma patients did worse in comparison with high-grade urothelial carcinoma and, when separated, carcinosarcoma patients did worse than sarcomatoid variant of urothelial carcinoma patients. Commenting on the aforementioned database results, Amin [15] stated that a potential well-known draw-back of analysing data from large publicly reported base is that the cases are not centrally reviewed by a group of dedicated pathologists and that differing criteria and nomenclature may be used by different reporting pathologists. Some authors [20, 22] stated that there is the likelihood that as many leading centre classification schemes and papers have adopted and endorsed a unifying approach to terminology, this could have likely influenced and compounded the analysis of data which was evaluated without ascertaining if uniform criteria were used without a histological rereview of the study.

Amin [15] stated the following.

- The sarcomatoid areas (obvious sarcomatoid overgrowth) may merge with foci of urothelial carcinoma, squamous cell carcinoma, adenocarcinoma, or small-cell carcinoma and most commonly resemble a high-grade sarcoma, which has not been otherwise specified to have malignant fibrous histiocytoma histology.
- Heterologous differentiation may be present. However, this has no definite prognostic significance.
- In decreasing order of frequency, areas of osteosarcoma, chondrosarcoma, rhabdomyosarcoma, liposarcoma, angiosarcoma, or a mixture of sarcoma histologies may be seen in sarcomatoid urothelial carcinoma.
- In the absence of an obvious invasive carcinoma (urothelial, glandular, small cell, and so on), in a primary spindle cell tumour of the urinary bladder, a history of prior urothelial neoplasia, coexistence of in situ disease such as urothelial carcinoma in situ or strong and relatively diffuse cytokeratin immunoreactivity would be of help in establishing the diagnosis of sarcomatoid carcinoma over a primary sarcoma.

Shah et al. [23] reported that earlier treatment with radiation therapy and intravesical cyclophosphamide chemotherapy and external beam radiotherapy for carcinoma of the prostate gland had been associated with sarcomatoid carcinoma of the urinary bladder.

Some authors [24, 25] stated that the differential diagnosis of sarcomatoid variant of urothelial carcinoma includes benign or locally aggressive conditions and some of these conditions include: pseudosarcomatous myofibroblastic proliferations (post-operative spindle cell nodules) and pseudo-tumours (inflammatory myofibroblastic tumours) of the urinary bladder, urothelial carcinoma with chondroid or osseous metaplasia (e.g., the absence of atypical cartilage or osteoid, resp.), primary sarcomas, mainly leiomyosarcomas.

Some authors [6, 15] stated that pseudosarcomatous myofibrosarcomatous proliferations may form large mass lesions which protrude in the lumen of the urinary bladder as a polypoid tumour and/or widely involve the urinary bladder wall including the muscularis propria. High cellularity, frequent mitoses (not atypical), and necrosis together with an infiltrative growth compound the distinction with sarcomatoid carcinoma. The lesion often has a myxoid background with granulation-tissue-type vascularity, extravasated red cells, and an inflammatory infiltrate. They [6, 15] also stated that a zonal pattern of distribution, that is, more myxoid and hypocellular regions toward the surface and greater cellularity with a fibrous background toward the base, a “nodular-fascitis”-type appearance of the lesion, the absence of an epithelial component as well as the absence of nuclear atypia (hyperchromasia, chromatin abnormalities, and anaplasia) are key in the distinction from a malignant process. In addition, Jones and Young [6] stated that a subgroup of sarcomatoid carcinomas may exhibit a more prominent myxoid background which may add to the marked diagnostic overlap and to the difficulty in distinguishing between the two entities. Amin [15] had an anecdotal experience, which had to some degree also been commented upon in the literature, that leiomyosarcomas of the urinary bladder also have a more prominent myxoid appearance.

Some authors [25] stated that immunohistochemical studies, similar to morphology, have marked overlap in the staining characteristics between pseudosarcomatous myofibroblastic proliferations, sarcomatoid carcinoma, and leiomyosarcomas, even though they had recently observed that the use of a judicious panel of immunostaining agents (pan-cytokeratin, smooth muscle actin, desmin, Alk-1, p63, CK5/6, and/or high-molecular-weight cytokeratin) interpreted strictly within the morphological context may be of some value. It has also been stated that

- pseudosarcomatous myofibroblastic proliferations are usually positive for pan keratin, smooth muscle actin, desmin, and alk-1;
- sarcomatoid carcinomas may also stain positively for pan-cytokeratin and smooth muscle actin and rarely with desmin, but they may be distinguished by their positivity for p63, CK5/6, and high-molecular-weight cytokeratin (in 10% to 40% of cases);
- leiomyosarcomas are positive for actin, desmin (usually extensively), and occasionally pan-cytokeratin (usually weak or focal). They are negative for p63, CK5/6, high-molecular-weight cytokeratin, and Alk-1 [15, 26].

Amin [15] iterated that the differential immunostaining characteristics and the extent of staining of the individual markers between these three differential diagnostic considerations are important in the ultimate weight of support immunohistochemistry provides in this difficult area.

Amin [15] stated that there are no standardized clinical management protocols for sarcomatoid carcinoma and that adjuvant therapy has varied from institution to institution and the treatment paradigms may be different from the therapy for a primary sarcoma, for example, leiomyosarcoma. Black et al. [3] reported down staging (PT0 at cystectomy) for almost half of patients in their centre. Amin [15] additionally stated that almost all the cases of sarcomatoid carcinomas present at a high stage; they often exhibit nodal and/or distant metastases, and they are associated with very poor prognosis. Information gained from a number of publications would indicate that an estimated 70% of patients with sarcomatoid variant of urothelial carcinoma die within 2 years of diagnosis [2, 4, 6, 11, 16–21, 27].

Amin [15] stated that in comparison with patients with urothelial carcinoma alone, patients with sarcomatoid variant of urothelial carcinoma are at a greater risk for death even after adjusting for the stage of the tumour at presentation.

Arun et al. [28] in 2008 reported a 40-year-old man who presented with abdominal mass of four months duration and haematuria of recent onset. His examination revealed a huge mass which involved the left half of his abdomen. He had a computed tomography scan which demonstrated the mass to be arising from the left kidney. He underwent cystoscopy which revealed polypoidal extension of the growth through the left ureteric orifice. He underwent left radical nephroureterectomy. Histological examination of the specimen revealed sarcomatoid variant of urothelial carcinoma. Arun et al. [28] reiterated statements made by other authors and stated the following.

- Sarcomatoid carcinoma is one of the rare variants of urothelial carcinoma and sarcomatoid carcinoma involving the renal pelvis is rarer still.
- Histological differentiation from true sarcoma is difficult and immunohistochemistry is helpful in this regard.
- Generally sarcomatoid carcinomas present at a high stage and have a poor prognosis.

Sarkissian and Lara [29] reported a 52-year-old man who presented with left flank pain, haematuria, nausea, vomiting, and fever. He had a past medical history of superficially invasive, low-grade papillary urothelial carcinoma and Gleason 6 adenocarcinoma of prostate for which he had undergone cystoprostatectomy with ileal conduit four years earlier. Histological examination of the specimen revealed that all the margins of the specimen were clear of tumour. His investigations revealed an elevated white blood cell count (23,000) and elevated creatinine (3.8) and computed tomography scan revealed findings which were adjudged to be consistent with an abscess of the left kidney. The kidney was nonfunctional based upon renal scan. The aetiology of this process was unclear; there was no sign of obstruction at the ureteroileal anastomosis. Pursuant to percutaneous nephrostomy with drainage of “this abscess”, there was no clinical improvement observed. He therefore underwent surgical exploration and nephrectomy. Macroscopic examination of the specimen revealed multiple irregular fragments of pink-tan, focally haemorrhagic partially soft, and partially firm tissue with areas consistent with necrotic renal cortex, medulla, pelvis, calyces, and perinephric fat. There was also grey-white, friable and necrotic tissue present in the morcellated fragments. Histological examination of the specimen revealed a biphasic malignant neoplasm with epithelial and sarcomatoid elements. The sarcomatous portion of the tumour consisted of sheets of malignant spindle cells with large vesicular nuclei, prominent nucleoli, and frequent mitotic figures. The tumour also had areas of frankly invasive squamous carcinoma with origin from the renal pelvis as well as low-grade papillary urothelial carcinoma. The tumour contained myxoid areas and giant cells. The residual renal parenchyma was extensively necrotic, and there was abscess formation and diffuse glomerular sclerosis. Immunohistochemical staining of the specimen revealed biphasic expression of the sarcomatous component with strong positivity for vimentin and focal positivity for keratin AE1/3 [29]. Sarkissian and Lara [29] concluded that (a) high-grade transitional cell carcinoma can imitate severe purulent kidney infection, (b) this disease is characterized by an unfavourable course and poor prognosis, (c) in spite of the clinical signs of inflammatory renal disease, an underlying neoplastic disorder should always be considered, especially in patients with prior history, and (d) in uncertain cases, a quick preoperative biopsy and histological examination of the kidney are recommended.

Leder and Dunnick [30] stated that malignant tumours from the urothelium of the renal pelvis account for only 5% of urinary tract neoplasms, with the most common of these being transitional cell carcinoma and squamous cell carcinoma. Cohen and Johansson [31] stated that of the tumours that arise from the renal pelvic urothelium, approximately 90% are transitional cell carcinomas.

Piscioli et al. [32] in 1984 reported the first case of sarcomatoid carcinoma of the renal pelvis. They concluded that the tumour should be diagnosed as sarcomatoid carcinoma and they should be discriminated from “true” carcinosarcoma.

Thiel et al. [33] stated that in some cases, the terminology carcinosarcoma is used as a synonym for sarcomatoid carcinoma but they are considered clearly separate entities. On the other hand, unlike sarcomatoid carcinoma, carcinosarcoma exhibits, in addition to a malignant epithelial component, specific features of mesenchymal differentiation such as chondrosarcoma, osteosarcoma, rhabdomyosarcoma, liposarcoma, or malignant fibrous histiocytoma [29]. Some authors [16, 22, 33, 34] stated that histological distinction of sarcomatoid carcinomas from carcinosarcomas is often difficult and immunohistochemistry is a helpful diagnostic adjunct in the correct diagnosis.

A number of authors [5, 32, 34, 35] had stated that sarcomatoid carcinoma of the kidney is usually a variant of renal cell carcinoma; nevertheless, transitional cell carcinoma of the renal pelvis might also assume a sarcomatoid appearance, even though this occurs only rarely. Other authors [36, 37] had stated that the sarcomatoid renal pelvic tumour should not be confused with sarcomatoid renal cell carcinoma, a high-grade malignant variant of renal parenchymal origin. Perez-Montiel et al. [38] stated the following.

- Demonstration of a transitional cell carcinoma component should be important in the differential diagnosis.
- The possibility of a high-grade urothelial carcinoma should always be considered in the evaluation of a tumour displaying unusual morphologic features in the renal pelvis, and attention to proper sampling as well as the use of immunohistochemical stains will be of importance to arrive at the correct diagnosis.

Wallach et al. [39] reported a 67-year-old woman who had experienced several weeks of visible haematuria with clots. She underwent elsewhere cystoscopy and transurethral resection of a urinary bladder tumour and histology of the resected tumour revealed a high-grade urothelial carcinoma of the urinary bladder with areas of sarcomatoid and neuroendocrine differentiation. She was advised to undergo radical cystectomy. She had a repeat cystoscopic biopsy from her urinary bladder and histological examination of the specimen revealed invasive high-grade urothelial carcinoma with prominent sarcomatoid and neuroendocrine elements, and invasion of the muscularis propria and the tumour was staged as T2AN0. The patient was further advised that bladder-conserving approaches were not recommended in view of the likely poor response to chemoradiation by the sarcomatoid elements, rendering radical cystectomy the standard of care. Nevertheless, the patient continued to seek bladder preservation and presented to a different hospital to discuss bladder-sparing options. At this institution she was also advised that radical cystectomy was the traditional standard of care, but it was agreed that chemoradiation was a reasonable alternative because cystectomy could be reserved for salvage operation. At this institution her clinical examination did not reveal any significant finding. She denied further haematuria, increased urinary frequency, urinary urgency, urinary retention, dysuria, incontinence, anorexia, or weight loss. She stated that she was a nonsmoker but she reported a significant history of second-hand exposure to smoke. She had a past medical history of peptic ulcer and had in the past undergone appendicectomy and tonsillectomy. In this new institution, a treatment programme was developed for her, which consisted of 7 weeks of weekly radiation therapy integrated with chemotherapy, with follow-up routine cystoscopy thereafter to monitor response. Her radiation programme consisted of 36 fractions over 49 days, with a dose of 39.6 Gy to the bladder and pelvic lymph nodes followed by 25.2 Gy to the bladder tumour. Her chemotherapy regimen was comprised of cisplatin 35 mg/m^2^ (49 mg in 500 mL normal saline over 1 hour) at the beginning of weeks 1, 3, 5, and 7, with each cycle divided over 2 consecutive days. She was also treated with palonosetron hydrochloride 0.25 mg intravenous bolus and dexamethasone 20 mg intravenous 50 mL saline while receiving chemotherapy. She tolerated the therapy well, without interruptions and with only mild diarrhoea which improved with loperamide. She underwent cystoscopy 2.5 months after chemoradiation which revealed no evidence of any cancer. Biopsies were taken from the initial location at that time and histological examination of the biopsies were negative for carcinoma [39]. Wallach et al. [39] stated that their report demonstrated only 1 case with limited follow-up but the favourable tumour response, coupled with an improved quality of life with a native bladder, provided a strong argument for bladder preserving chemoradiation as an alternative treatment regimen for sarcomatoid variant of urothelial carcinoma of the urinary bladder.

Lopez-Beltran et al. [2] as well as Sauer et al. [40] also reaffirmed statements made by other authors that sarcomatoid carcinomas of the urinary bladder are exceedingly rare and that they are highly aggressive spindle-cell neoplasms which are usually diagnosed at an advanced stage and they have a median survival of 10 months. Castelao et al. [41] iterated that radical surgery with adjuvant radiation therapy to eradicate microscopic disease is the traditional standard treatment for most sarcomas, in view of the fact that these tumours show poor response to primary radiation therapy. Wallach et al. [39] stated that the literature regarding nonsurgical therapy for these bladder tumours is scarce but one case report described a patient with metastatic sarcomatoid carcinoma of the urinary bladder who demonstrated a clinical complete remission after cisplatin and gemcitabine, and another described a complete remission pursuant to neoadjuvant chemotherapy using carboplatin and gemcitabine followed by partial cystectomy [42, 43].

Nomikos et al. [44] reported a 32-year-old woman with neurogenic bladder due to spina bifida who had been managed with intermittent self-catheterizations since puberty. She presented with visible haematuria and recurrent urinary tract infections. She did not have any family history of bladder cancer. She underwent cystoscopy which revealed a bulky solid mass occupying the posterior bladder wall. She had transurethral resection biopsies of the tumour and histological examination of the tumour revealed muscle-invasive sarcomatoid urothelial carcinoma with carcinoma in situ of the bladder neck. She had computed tomography scan which did not show any evidence of nodal disease or distant metastasis. She underwent radical cystectomy with anterior exenteration and ileal conduit formation. Microscopic examination of the specimen revealed that the tumour was a high-grade urothelial carcinoma, Grade III according to the classification of the World Health Organization (WHO) of 1973, invading the whole bladder wall and pericystic lipoid tissue (stage pT3a). A large subset of the tumour assumed spindle cell/sarcomatoid appearance with high mitotic rate and atypical mitoses. Immunohistochemical staining of the tumour revealed that the tumour cells were positive for the epithelial membrane antigen (EMA), cytokeratin 18 (CK18), and vimentin. Some tumour cells were positive for CD117 (c-kit), whereas all of them were negatively stained for CK7, CK20, Desmin, and CD34. Nomikos et al. [44] stated that the aforementioned histologic and immunohistochemical evidence of both epithelial and mesenchymal differentiation would classify this tumour as sarcomatoid variant of urothelial carcinoma without heterologous elements. The patient had completed 4 cycles of gemcitabine and carboplatin and at 9 months of follow-up she remained disease free. Nomikos et al. [44] stated the following.

- Their case was the first report of bladder urothelial carcinoma of the sarcomatoid variant diagnosed in a young patient with spina bifida.
- High index of clinical suspicion and appropriate usage of immunohistochemical techniques are essential for fast diagnosis of this rare clinical entity.
- Primary radical cystectomy and adjuvant chemotherapy may improve outcome.

Bostwick and Cheng [45] stated that sarcomatoid carcinoma is a biphasic malignant neoplasm which exhibits morphologic and/or immunohistochemical evidence of epithelial and mesenchymal differentiation. By means of immunohistochemistry, epithelial elements react with cytokeratins, whereas stromal elements react with Vimentin or specific markers corresponding to the mesenchymal differentiation. Microscopically, it is composed of spindle cell/sarcomatoid elements with high mitotic rate and atypical mitoses.

Wang et al. [46] undertook a retrospective review of their experience in managing patients with sarcomatoid bladder cancer between 1997 and 2011 in order to better define the behaviour and outcomes of the disease. Wang et al. reported that the median age of the patients was 63 years. All of the patients presented with high-grade histology. 85% of the patients presented with muscle-invasive disease and 50% presented with stage IV carcinoma. Ten of 14 (71%) patients underwent cystectomy. They reported that patients with sarcomatoid variant of urothelial carcinoma were younger (*P*, 0.010), and they more commonly presented with high-grade histology (*P*, 0.01) and advanced stage disease (*P*, 0.01), in comparison with patients who had conventional urothelial carcinoma. Wang et al. [46] also reported that at a median follow-up of 7 months (range 1.3 to 112 months), five patients (35.7%) had died in the follow-up; the two-year survival was 53.5%. They also reported three patients with long-term survival. Wang et al. [46] stated that sarcomatoid bladder cancer was associated with poor prognosis and that multimodality therapy may improve the outcome of the patients. Wang et al. [46] stated the following.

- Their study showed patients with this rare form of urothelial carcinoma are diagnosed at a younger age and they present with a higher grade of histologic malignancy as well as an advanced stage in comparison with patients without sarcomatoid differentiation.
- Consistent with previous studies, the cancer specific survival of this cohort of carcinosarcoma of the urinary bladder was poor.
- In view of the absence of randomized and controlled trials, there is no standard treatment for this disease.
- Only few studies reported the use of chemoradiotherapy and chemotherapy after surgical resection of carcinosarcoma of the urinary bladder.
- In their series, aggressive multimodality treatment, in 3 patients, led to complete responses and markedly improved survival. And it was gratifying to report the long-term survival, multiple years, of the three patients with sarcomatoid bladder cancer.
- The low incidence of sarcomatoid variant of urothelial carcinoma renders the conduct of randomized trials rather impossible and drawing clear guidelines for its management is subsequently difficult. However, their series would suggest that long-term survival is possible in patients treated with multimodality therapy, and the optimal treatment modality has yet to be defined.
- Future studies must investigate the combination of chemotherapy with new targeted therapies.

Arita et al. [47] stated that the cytological features of sarcomatoid variant of urothelial carcinomas are not well known and only one cytological analysis of sarcomatoid variant of urothelial carcinoma had previously been reported. Arita et al. [47] reported their study which included the first analysis of cytological features from a series of sarcomatoid variant of urothelial carcinoma and discussed possible differential diagnostic considerations. They analysed and reviewed the cytological features of a series of sarcomatoid variant of urothelial carcinoma cases which included 6 voided urine specimens from 3 patients with sarcomatoid variant of urothelial carcinoma. They reported that several characteristic cytological features were revealed and some of these include the following.

1. Tumour cells were abundant in a necrotic background and while single tumour cells were predominant, small clusters of cells were occasionally present.
2. Tumour cells were large-sized and round to polygonal in shape with ill-defined cell borders.
3. Tumour cells had a high nuclear/cytoplasmic ratio and enlarged round to oval nuclei containing coarse chromatin and occasional nucleoli.
4. Spindle-shaped atypical cells were rarely identified (1/6 specimens).

Arita et al. [47] stated that the cytological features of 1, 2, and 3 are indistinguishable from those of conventional invasive high-grade urothelial carcinoma. Arita et al. [47] postulated that these tumour cells originated from the conventional high-grade urothelial carcinoma component of sarcomatoid variant of urothelial carcinoma and this component is usually present in this type of lesion, particularly on the surface of the tumour. Moreover, the sarcomatoid component of sarcomatoid variant of urothelial carcinoma is usually present in the deeper portion of the tumour, and therefore detection of this component in the voided cytological specimen is low. Even though cytodiagnosis of sarcomatoid variant of urothelial carcinoma is extremely difficult, cytodiagnosis of malignancy may prove possible due to the presence of a conventional urothelial carcinoma component.

## 4. Conclusions

Sarcomatoid variant of urothelial carcinoma is a rare aggressive type of tumour which tends to present at a younger age and at a higher grade and stage.

Sarcomatoid variant of urothelial carcinoma is associated with inferior outcome in comparison with conventional urothelial carcinoma.

There is anecdotal information which would suggest that chemoradiation in addition to radical surgery may improve prognosis of the tumour.

There is a need for a multicentre trial of various treatment options for sarcomatoid variants of urothelial carcinoma in order to arrive at a consensus opinion regarding the best treatment option.

Urologists and oncologists should be encouraged to report cases of sarcomatoid variant of urothelial carcinoma they encounter in order to contribute to the understanding of the biological behaviour of the tumour.
